# Atypical lung carcinoid with prominent mucinous stroma: unusual pathologic finding in pulmonary carcinoid

**DOI:** 10.1093/omcr/omag020

**Published:** 2026-03-23

**Authors:** Ghaida S AlSugair, Wajd A Althakfi

**Affiliations:** Department of Basic Sciences, College of Medicine, Princess Nourah Bint Abdulrahman University, Airport Road, P.O. Box 84428, Riyadh 11671, Kingdom of Saudi Arabia; Histopathology Unit, Department of Pathology, King Saud University, Diriyah Road, P.O. Box 2455 Riyadh 11451, Kingdom of Saudi Arabia

**Keywords:** pulmonary carcinoid, neuroendocrine tumor, mucinous stroma, Thymic neoplasm, lobectomy, prognosis, case report

## Abstract

Background: Atypical carcinoids (ACs) are uncommon neuroendocrine tumors of the lung, and the presence of a prominent mucinous stroma is an exceptionally rare feature that may complicate diagnosis. Highlighting such variants is important for avoiding misclassification with mucin-producing pulmonary tumors. Case Presentation: A 34-year-old nonsmoking female presented with mild chest pain, minimal cough, and night sweats. Imaging revealed a well-circumscribed right middle-lobe lesion measuring 4.1 cm, and bronchoscopy showed a white-tan, fleshy mass filling the bronchial lumen. Pathology Findings: Histologic examination demonstrated a well-defined neoplasm with organoid, trabecular, follicular, pseudopapillary, and solid patterns and striking mucinous stromal change. Immunohistochemical staining was positive for CK AE1/AE3, synaptophysin (diffuse), chromogranin (diffuse), and TTF1, and negative for CK7, CK20, and CDX2. Focal necrosis and a mitotic rate of up to 5/2 mm^2^ supported the diagnosis of AC. Conclusion: This case represents a rare morphologic variant of AC distinguished by a prominent mucinous stroma, a feature that may obscure the diagnosis by simulating other mucin-producing lung tumors. Recognizing this uncommon pattern is crucial, as it directly impacts diagnostic accuracy and subsequent clinical decision-making.

## Introduction

Carcinoid tumors of the lung account for about 2% of all lung cancers (ratio of typical to atypical is 10:1), arising from the enterochromaffin cells lining the aerodigestive tract [1]. They are divided into two types: typical carcinoid tumors and atypical carcinoid tumors, which are low- and intermediate-grade neuroendocrine tumors, respectively. Approximately 80% of pulmonary carcinoids arise centrally, while 20% arise peripherally. According to the Thoracic WHO classification 5th edition [1], pulmonary carcinoids are classified as typical or atypical based on the number of mitotic figures per 2 mm^2^ and the presence of necrosis. 1 Typical carcinoids have fewer than 2/2 mm^2^ mitoses and are without necrosis, while ACs have 2-10/2 mm^2^ mitoses and/or necrosis and have focal, punctate, or comedo-like morphology [1].

However, both carcinoid subtypes can have a broad array of histological patterns, including organoid, trabecular, palisading, papillary, pseudo-glandular, rosette-like, spindle, and follicular growth. Classically, the stroma of carcinoids is richly vascularized and can exhibit hyalinization, bone formation, or cartilage formation. In the literature, cases of stromal amyloid and a prominent mucinous stroma have been reported [2].

Having such a heterogeneous morphology, carcinoid tumors can be muddled with other primary and metastatic tumors and require careful morphological and immunohistochemical analysis for differential diagnosis [1].

Here, we present a rarely reported case of AC with a protuberant mucinous stroma. We also performed a detailed histopathologic and immunohistochemical examination to rule out other primary and metastatic mucin-rich tumors.

## Case report

**Figure 1:**
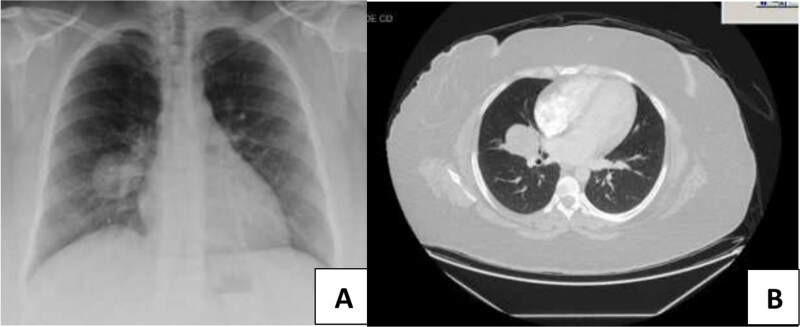
Radiological examination of the chest of the patient. (A) Chest X-ray revealed a demarcated nodule located in the middle right lobe. (B) Chest CT scan displayed a 4.1 cm nodule with no mediastinal lymph node enlargement.

A 34-year-old nonsmoker female with a nonremarkable previous medical history presented with mild chest pain, minimal cough, and night sweats. There was no history of lung or gastrointestinal cancer in her family. The patient was referred to the thoracic surgery clinic due to an incidental finding of a right lung middle lobe mass revealed by a computed tomographic (CT) scan of the chest, which was done as pre-operative imaging before hernial repair surgery. On standard chest X-ray and CT scan, a defined nodule was visible in the middle right lobe of the lung (4.1 cm in diameter) (Fig. 1).

**Figure 2:**
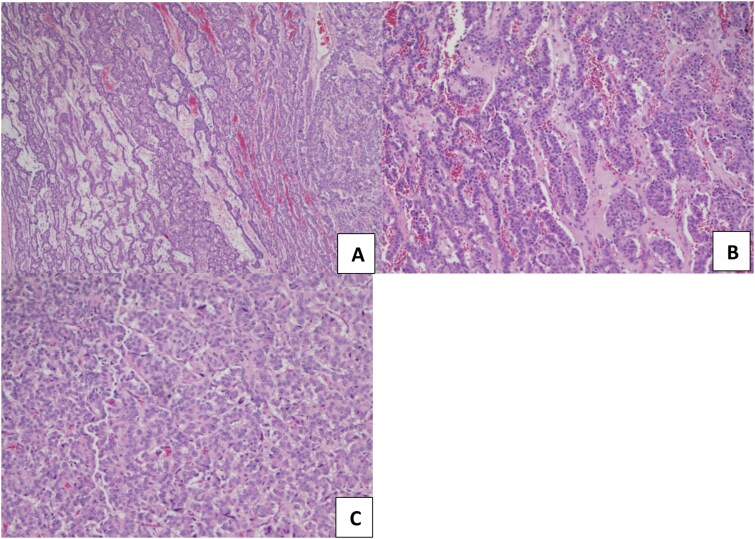
Histopathological analysis of atypical lung carcinoid with different proliferation patterns observed under a light microscope and after H&E stain. (A, B) trabecular proliferation pattern with vascular stoma, original magnification x20 (C). Solid and organoid proliferation patterns, original magnification x40.

**Figure 3:**
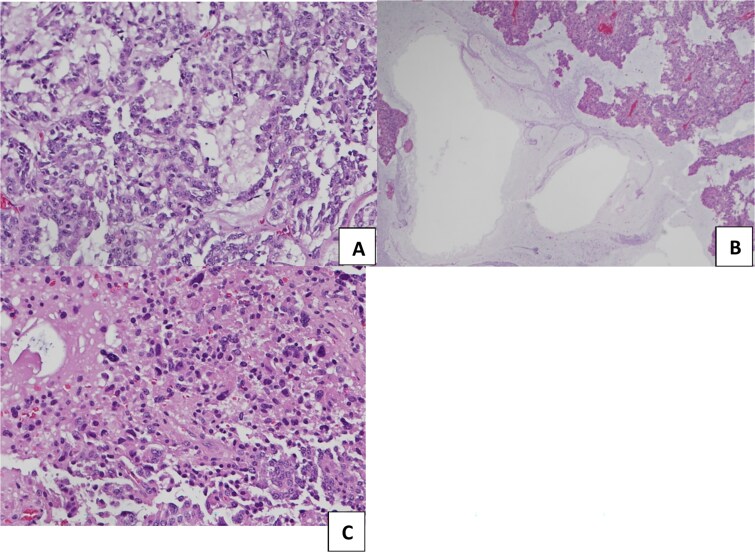
(A, B) abundant mucinous stroma, original magnification x40. (C) Area of necrosis, original magnification x100.

The patient underwent a right middle lung lobectomy. On gross examination, a round to oval white-tan well-demarcated mass filled the bronchial lumen, measuring 4.2 × 3.3 × 3.0 cm, which was 2 mm away from the uninvolved bronchial margin. Grossly, the lesion had a tan, fleshy cut surface. On histopathological examination, the tumor exhibits various growth patterns: organoid, trabecular, follicular, pseudopapillary, and solid patterns (Fig. 2). The cells show neuroendocrine differentiation with salt-and-pepper chromatin and mild eosinophilic cytoplasm. The classic stroma of high vascularization was seen, but the striking diffuse finding was the abundant mucinous stroma. Multiple small foci of necrosis were present, and the mitotic rate was five mitoses per 2 mm^2^ in the hotspot (Fig. 3).

**Figure 4:**
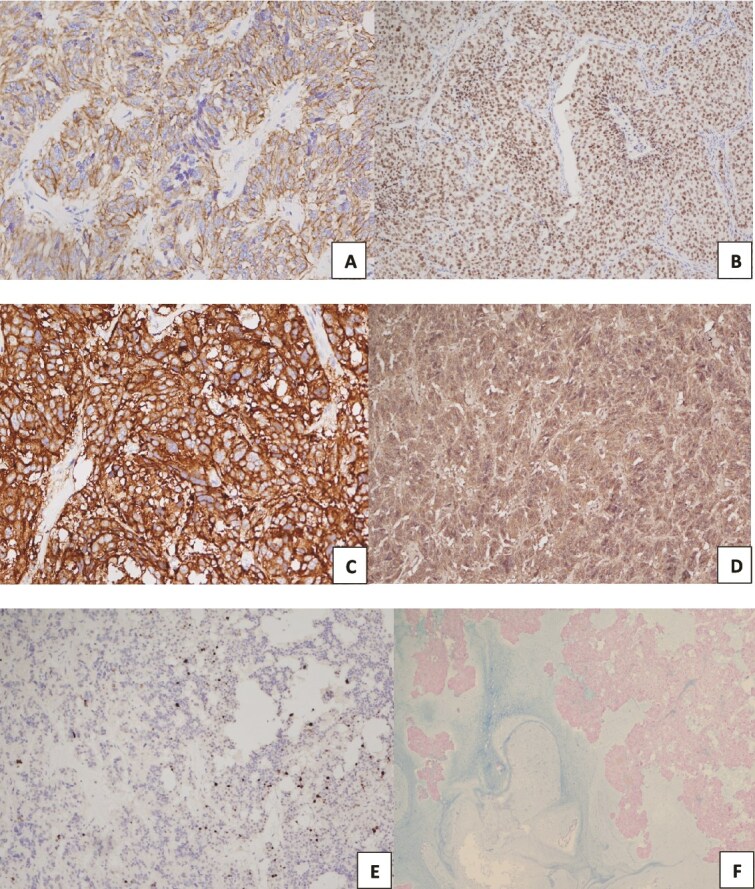
Tumor cells are positive for pan-CK, TTF-1, Synaptophysin, and chromogranin (A-D, respectively). The staining index for Ki67 was between 5 and 20% (E). Alcian blue stain demonstrates extracellular mucinous secretions. (original magnification x40 (F).

On immunohistochemistry, the tumor showed diffuse positivity for pan-cytokeratin (CK AE1/AE3) and the neuroendocrine markers synaptophysin and chromogranin, with additional positivity for thyroid transcription factor 1 (TTF1). In contrast, CK7, CK20, and caudal-type homeobox 2 (CDX2) were negative. The Ki-67 proliferation index was approximately 10% (Fig. 4). The detailed immunohistochemical findings are summarized in Table 1. These findings, in combination with the histologic features, support the diagnosis of atypical carcinoid with prominent mucinous stroma.

**Table 1:** Immunohistochemical findings.

Marker	Result	Notes
CK AE1/AE3	Positive	—
Synaptophysin	Positive	Diffuse
Chromogranin	Positive	Diffuse
TTF-1	Positive	—
CK7	Negative	—
CK20	Negative	—
CDX2	Negative	—
Ki-67	~10%	Proliferation index

## Discussion

Pulmonary carcinoid tumors are malignant neuroendocrine neoplasms that generally follow an indolent course, yet they can display a wide range of histologic patterns. Carcinoids with abundant mucinous stroma are exceedingly rare, with only two well-documented pulmonary atypical carcinoid cases reported in the literature [3, 4]. Extrapulmonary counterparts—particularly in the thymus have been reported more frequently, with at least six published cases demonstrating similar mucin-rich stromal features [5, 6]. Many of these thymic cases exhibited destructive growth, early metastasis, or poor outcomes, suggesting that mucinous stromal production may correlate with more aggressive biological behavior.

Beyond case reports, mucinous stromal change has been described in a subset of extrapulmonary neuroendocrine neoplasms, including pancreatic and gastrointestinal NETs, where mucin deposition has been associated with altered tumor–stroma signaling and more infiltrative growth patterns [7, 8]. Although these findings cannot be directly extrapolated to pulmonary carcinoids, they support the concept that stromal mucin in NETs may reflect underlying biological alterations rather than a purely degenerative process.

The biological significance of mucinous stroma in neuroendocrine tumors remains uncertain. Proposed mechanisms include stromal metaplasia, mucin-secreting transdifferentiation, or tumor–stroma signaling pathways that promote extracellular mucin deposition. Recent molecular studies have emphasized the importance of extracellular matrix remodeling in NET progression, including mucin-associated pathways such as MUC1/MUC2 dysregulation and TGF-β–mediated stromal activation [9, 10]. Given the limited number of reported cases, firm conclusions regarding the biological impact of mucinous stroma in atypical carcinoids cannot yet be drawn.

From a diagnostic perspective, mucin-rich atypical carcinoids require careful distinction from primary mucinous lung tumors, including mucinous adenocarcinoma, mucin-rich salivary-type tumors, and mucinous cystadenoma, as well as metastatic mucin-producing neoplasms [11]. In addition, rare mucin-producing salivary gland-type pulmonary tumors such as mucoepidermoid carcinoma and adenocarcinoma NOS should be considered [12]. Correlation of clinical, radiologic, histologic, and immunophenotypic features remains essential for accurate classification.

Management of pulmonary carcinoids continues to rely on complete surgical resection. For atypical carcinoids, lobectomy with systematic lymph node sampling is preferred. Given the aggressive behavior described in mucin-rich thymic carcinoids, careful assessment of lymph nodes and margins may be warranted in similarly unusual pulmonary variants. Adjuvant therapy remains controversial; ENETS recommends postoperative therapy for node-positive ACs, while NCCN suggests it for stage III disease [13]. More recently, ESMO guidelines have emphasized individualized risk stratification incorporating mitotic index, Ki-67, margin status, and nodal disease when considering adjuvant treatment in pulmonary NETs [14].

Prognostic uncertainty remains significant due to the rarity of mucin-rich ACs. Lung cases have shown variable behavior, whereas thymic cases demonstrate poorer outcomes. Although stromal mucin has been proposed as a possible marker of biologic aggressiveness, current evidence is insufficient and inconsistent across organ systems [7–10]. As such, the prognostic impact of mucinous stroma cannot be reliably established and warrants documentation and long-term follow-up.

We have described an unusual and rarely reported variant of atypical carcinoid with prominent mucinous stroma in the lung. The tumor’s behavior is not well characterized, and the subsequent optimal treatment options remain uncertain. Similar cases in the thymus exhibit aggressive behavior and outcomes. We also emphasize the importance of clinicopathologic and radiologic evaluation to ensure accurate diagnosis and appropriate management.
